# Hydrogen Production by Steam Reforming of Ethanol and Dry Reforming of Methane with CO_2_ on Ni/Vermiculite: Stability Improvement via Acid or Base Treatment of the Support

**DOI:** 10.3390/molecules29112575

**Published:** 2024-05-30

**Authors:** Hanane Mahir, Abdellah Benzaouak, Farah Mesrar, Adnane El Hamidi, Mohamed Kacimi, Luca Consentino, Leonarda Francesca Liotta

**Affiliations:** 1 Laboratory of Nanomaterials, Nanotechnologies and Environment, Physical-Chemistry of Materials, Catalysis and Environment Unity, Department of Chemistry, Faculty of Sciences, Mohammed V University in Rabat, Avenue Ibn Battouta, BP:1014, Rabat 10000, Morocco; hanane_mahir@um5.ac.ma (H.M.); mesrar.farah@gmail.com (F.M.); m_kacimi2000@yahoo.fr (M.K.); 2 Laboratory of Spectroscopy, Molecular Modelling, Materials, Nanomaterials, Water and Environment, Environmental Materials Team, ENSAM, Mohammed V University in Rabat, B.P. 6207 Avenue des Forces Armées Royales, Rabat 10100, Morocco; a.elhamidi@um5r.ac.ma; 3 Istituto per lo Studio dei Materiali Nanostrutturati (ISMN)-CNR, Via Ugo La Malfa, 153, 90146 Palermo, Italy; lucaconsentino@cnr.it

**Keywords:** dry reforming, steam reforming, nickel catalyst, vermiculite, clay, mixed oxide

## Abstract

In this study, vermiculite was explored as a support material for nickel catalysts in two key processes in syngas production: dry reforming of methane with CO_2_ and steam reforming of ethanol. The vermiculite underwent acid or base treatment, followed by the preparation of Ni catalysts through incipient wetness impregnation. Characterization was conducted using various techniques, including X-ray diffraction (XRD), SEM–EDS, FTIR, and temperature-programmed reduction (H_2_-TPR). TG-TD analyses were performed to assess the formation of carbon deposits on spent catalysts. The Ni-based catalysts were used in reaction tests without a reduction pre-treatment. Initially, raw vermiculite-supported nickel showed limited catalytic activity in the dry reforming of methane. After acid (Ni/VTA) or base (Ni/VTB) treatment, vermiculite proved to be an effective support for nickel catalysts that displayed outstanding performance, achieving high methane conversion and hydrogen yield. The acidic treatment improved the reduction of nickel species and reduced carbon deposition, outperforming the Ni over alkali treated support. The prepared catalysts were also evaluated in ethanol steam reforming under various conditions including temperature, water/ethanol ratio, and space velocity, with acid-treated catalysts confirming the best performance.

## 1. Introduction

In recent decades, mining activities have experienced significant growth, driven by advances in technology. Paradoxically, these mining operations have become major generators of waste on a large scale, necessitating efficient waste management strategies. In this context, one avenue for addressing the pollution stemming from these activities and adding value to mining operations is the valorization of this waste as catalysts [1].

The concept of a heterogeneous catalyst for various reactions involves the combination of an active phase deposited on a support. This support provides the catalyst with porosity, mechanical resilience, and facilitates the even dispersion of the active component [2]. Consequently, the support must possess a high specific surface area, and in some cases, it may play a pivotal role in the reaction mechanism, displaying catalytic activity itself, which is often referred to as bifunctional behavior.

In dry reforming of methane (DRM), the nature of the species involved, the dispersion and size of the metal particles, and their interactions with the support significantly influence the catalytic performance [3]. Alkaline and/or alkaline earth metals, despite lacking direct catalytic activity, often impact the acidity and basicity of the catalyst. Additionally, rare earth oxides help minimize carbon deposition and the sintering of active metals. Transition metals (Fe, Co, Ni, Cu) are commonly employed as catalysts, supported on various materials like silica, alumina, and MgO, and exhibit good activity. However, a notable drawback is the deposition of carbonaceous materials, which typically occurs due to methane decomposition and CO disproportionation, leading to catalyst deactivation or destruction. Catalysts based on noble metals (Pt, Pd, Ru, etc.) demonstrate high selectivity in catalytic methane reforming and are less prone to carbon deposits [3,4,5,6]. However, their elevated cost diminishes their economic appeal.

Research efforts have focused on developing new catalysts that resist carbon formation while maintaining cost effectiveness and availability. The substitution of conventional nickel catalysts supported on traditional oxides such as Al_2_O_3_, SiO_2_, and MgAl_2_O_4_ [7,8] with Ni-loaded hydroxyapatite, fluorapatite, and precipitated natural phosphate, showed promising results, as demonstrated by several studies conducted by our team [9,10].

The ongoing quest for diversifying catalytic material resources for economic and environmental reasons has piqued considerable interest. For instance, the use of natural clay as a support or catalyst has gained attention [11]. Furthermore, solid materials derived from mining residues are rich in components typically employed in catalysis. Utilizing mining materials as catalysts or supports presents a compelling alternative to traditional materials such as silica and alumina, primarily due to their low cost, ready availability, and permeability [11]. Other advantages include ease of shaping and the adaptability to specific application requirements. The potential for chemical modification of clays further enhances their utility across a range of technological applications, thereby adding value to this abundant natural resource [11,12,13].

In this context, we present the development of a novel catalyst derived from mining residues with negative value. Specifically, we report on the synthesis of an Ni catalyst supported by expanded vermiculite that has undergone acid or base treatment. Vermiculite is an aluminosilicate clay mineral with a layered structure [12,13,14]. Both in its natural form and when expanded, vermiculite exhibits exceptional characteristics such as being lightweight, durable, and eco-friendly (it is chemically inert and completely non-toxic). It has found extensive use in heavy metal adsorption [13,14]. Although there are only a few reported applications of vermiculite in catalysis [14,15,16,17], its potential remains underexplored.

The main objective of this study is to evaluate the performance of expanded vermiculite loaded with nickel catalyst after acid or basic treatment. This novel substrate was assessed in the processes of carbon dioxide reforming of methane [16,17,18] and steam reforming of ethanol, particularly under low-temperature conditions. The catalysts were synthesized and comprehensively characterized by means of various techniques such as XRD, FTIR spectroscopy, SEM–EDS, TG-DT analyses, and temperature-programmed reduction (H_2_-TPR). This investigation marks the inaugural phase of a series used for scrutinizing specific catalytic systems employed in the realm of hydrogen production.

## 2. Results and Discussion

### 2.1. Characterization

#### 2.1.1. X-ray Diffraction and Surface Area

**Figure 1:**
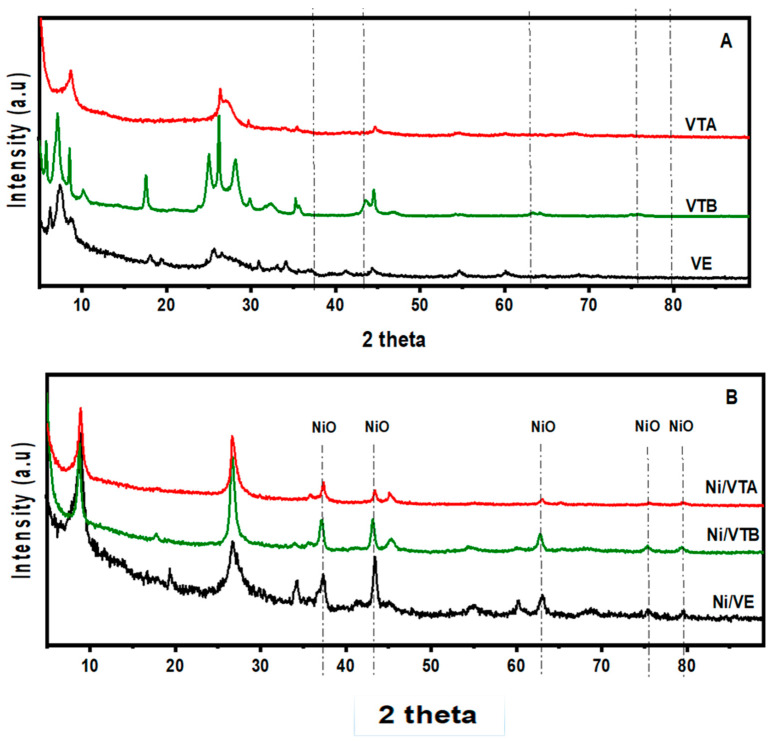
XRD powder patterns of the support materials (**A**) and of the loaded Ni catalysts (**B**).

Figure 1 shows the X-ray diffraction patterns for the vermiculite-based supports and the modified vermiculite-based Ni catalysts. As shown in Figure 1A, the initially expanded vermiculite sample (VE) displayed distinctive diffraction peaks at 2 theta angles of 6.24°, 7.45°, and 8.85°, indicating the presence of various layers of cations (Mg^2+^, Ca^2+^, Na^+^) within the vermiculite structure. In particular, the peak at 6.24° is typical of magnesium in the hydrated forms of vermiculite [17]; the 12.4 Å distance can be ascribed to the presence of partially dehydrated magnesium interlayer cations [18]; and the 8.85 Å spacing is related to interlayer potassium ions [17]. These observations highlight the diversity of cations present in different layers of the vermiculite structure.

Additionally, the existence of quartz impurities in the vermiculite is responsible for the peak observed at approximately 2θ = 25.6° [19].

Vermiculite exposed to 1 M HCl (VTA) showed significant changes in the XRD pattern compared to raw vermiculite (VE). Notably, only one peak at around 8.69° (10.24 Å) with low intensity was observed in VTA. The protonic form of clay is characterized by this feature. Acid treatment, which involves substituting interlayer Mg^2+^ and K^+^ cations with hydronium ions, leads to a reduction in the interlayer distance in the resulting vermiculite [19]. However, some reflections persisted at the high 2θ range, indicating that a fraction from the vermiculite was able to resist acid attack. This observation is consistent with the elemental analysis presented in Table 1, signifying that some of the interlayer potassium and magnesium ions were retained through this process.

Furthermore, the XRD patterns of vermiculite exposed to NaOH solution (VTB) displayed an increase in the intensity of characteristic peaks across the entire 2θ range compared to the starting material. This finding supports the notion that the alkali process performed at 60 °C was without substantial influence on the structure of the clay. Table 1, which reports the chemical composition mass (%), provides additional evidence in this regard. Indeed, the composition of the material after NaOH treatment (VTB) revealed no change compared to the raw material. Therefore, there was no leaching of the metal oxide species, suggesting that they were deposed–precipitated in situ [20]. In contrast, acid treatment of vermiculite significantly modified its chemical composition with removal of almost all of the Mg, Al, Fe, K, Ca, Na, and Ti oxides, with silica being predominant.

The X-ray diffraction (XRD) patterns of treated and untreated Ni-loaded catalysts (Figure 1B) displayed typical peaks of an NiO cubic structure at 2θ ≈ 37.34, 43.25, 62.82, 75.56, and 79.65°. Moreover, all of the obtained catalysts exhibited a new broad peak at approximately 26.8°, which is explained by the existence of amorphous silica [21]. The crystallite sizes were estimated according to the Debye–Scherrer equation. It was found that the average crystallite sizes of NiO in Ni/VTA and Ni/VTB were 17.1 nm and 18.3 nm, respectively, which are smaller compared to Ni/VE (21 nm).

Considering the variation in surface area (Table 1), a significant increase from 36 to 270 m^2^ g^−1^ was observed moving from the Ni/VE to the Ni/VTA catalyst, together with an increased pore volume (from 0.10 to 0.55 cm^3^ g^−1^). Conversely, no significant changes were noticed for the surface area and pore volume of Ni catalyst supported over the material treated with the NaOH solution. Both the Ni/VE and Ni/VTB samples showed a broad pore size distribution in the range between 5 and 30 nm, and only the Ni/VTA catalyst according to the increased silica oxide component showed a defined pore size distribution centered at 15 nm.

#### 2.1.2. SEM–EDS

**Figure 2:**
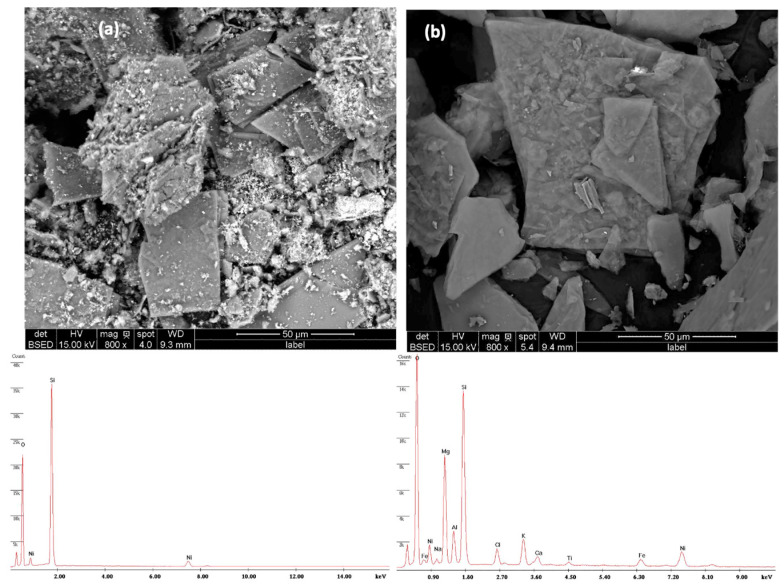
SEM micrographs and EDS spectra recorded for the Ni catalysts (**a**) Ni/VTA and (**b**) Ni/VTB.

Figure 2 displays SEM micrographs and EDS elemental analysis of the Ni-based catalysts treated by acid or base. The SEM images clearly show the structural changes in vermiculite following treatment with acid (Figure 2a) and base (Figure 2b), respectively. The surface is much rougher and more textured after the acid treatment, confirming the significant increase in specific surface area. This increase is crucial for the effective adsorption of reactive species. In contrast, the basic treatment led to a more homogeneous and less porous surface, suggesting significant molecular reorganization. In fact, during the acid treatment, vermiculite undergoes protonation, leading to the exchange of interlayer cations and the dissolution of some mineral components. This process can alter the surface acidity, textural properties, and chemical composition of the vermiculite. However, treatment with a base can result in deprotonation and hydrolysis reactions, which can influence the material’s surface charge, pore structure, and catalytic activity.

The addition of nickel as a catalyst to these modified supports showed promising catalytic performance. These aspects were also confirmed by EDS analysis.

#### 2.1.3. FTIR Spectroscopy

**Figure 3:**
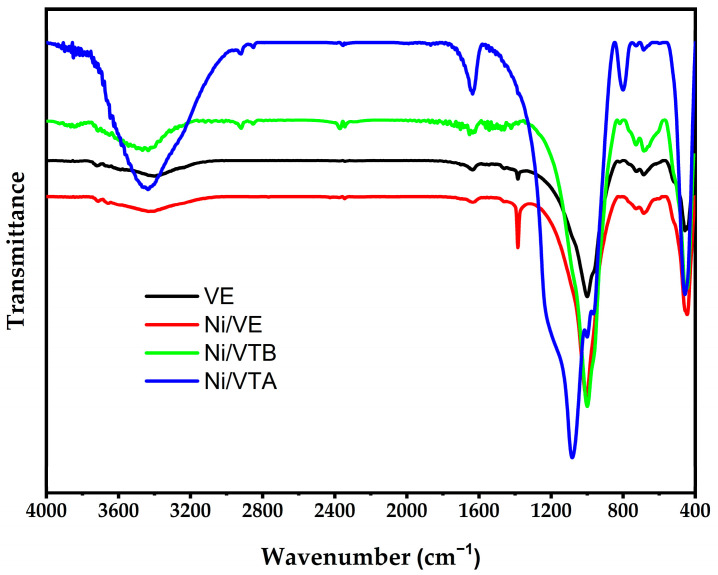
FTIR spectra of vermiculite (VE), prepared Ni/VE, Ni/VTB, and Ni/VTA catalysts.

Figure 3 presents the FTIR spectra of both expanded vermiculite and the resulting catalysts, highlighting several distinctive bands. The band observed at 3450 cm^−1^ is confidently assigned to OH stretching (νOH), providing evidence of hydrogen bonding between SiO-H surface groups. The band at around 1648 cm^−1^ can be attributed to the δOH vibrational mode of the H_2_O molecules bonded to the silica, as well as to the alumina. The νSi-O asymmetric stretching vibration of SiO_4_ is responsible of the bands observed at 1000 cm^−1^, while the in-plane bending is responsible for the bands observed at 1080 cm^−1^. Furthermore, a 668 cm^−1^ band is ascribed to Si-O deformation bands. Moreover, the band observed at 454 cm^−1^ is related to the vibrational mode of M-O-M (M = Si or Al). The 724.8 cm^−1^ band is assigned to the Al-O tetrahedral.

FTIR spectroscopy of Ni/VTA clearly displays a new peak close to 795 cm^−1^, which is assigned to the vibrational modes of SiO_4_ tetrahedra, very comparable to that observed for amorphous silica reported in the X-ray diffraction pattern. It is important to note that nickel addition into the supports does not seem to affect the IR spectra.

#### 2.1.4. TPR Analyses

**Figure 4:**
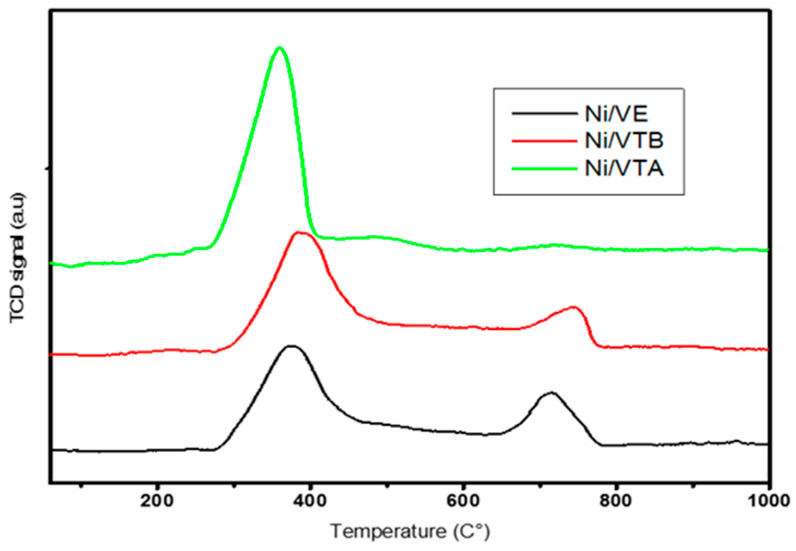
Profiles of H_2_-TPR for prepared Ni/VE, Ni/VTA, and Ni/VTB catalysts.

The reducibility of the Ni/VE, Ni/VTB, and Ni/VTA catalysts, as well as their interactions between the metallic species and the support, were investigated. Figure 4 illustrates the temperature programmed reduction (TPR) profiles. It is observed that the reductive process for all of the studied catalysts begins at a relatively low temperature of around 270 °C.

In the TPR profiles of Ni-supported expanded vermiculite and base-treated vermiculite, two distinct peaks were observed. The primary peak, located at 370 °C, was accompanied by a secondary hydrogen peak at higher temperatures, around 720 °C and 737 °C, respectively. This secondary peak can be ascribed to the reduction of NiOx species that exhibit high interactives properties with the support [22]. The Ni-supported vermiculite exposed to HCl solution showed a single main peak in its TPR profile at about 350 °C. This peak was attributed to free NiO being reduced to Ni dispersed on the support [23]. As suggested by this observation, the Ni species supported on the acid treatment vermiculite-based catalyst are more easily reducible. Furthermore, Ni/VTA TPR profiles resemble the profiles observed for Ni/SiO_2_ by Y.H. Taufiq-Yap et al. [24].

### 2.2. Catalytic Activity

#### 2.2.1. Dry Reforming of Methane

In this study, the catalytic performances in the dry reforming of methane by CO_2_ were evaluated and the effects of base and acid activation of vermiculite as support of the Ni catalyst were investigated. It is important to highlight that the catalysts were used without prior reduction, confirming enhanced catalytic activity; in contrast, the pre-treatment by H_2_ for reduction is commonly used to achieve comparable activity [25,26]. The active phase of the catalysts, NiOx, was reduced ‘in situ’ during the reaction using a mixture of CH_4_ and CO_2_. This methodology is consistent with other well-established studies [10,23,24]. This approach creates a durable and effective catalyst that can be used without the need for pre-reduction with H_2_ in dry reforming processes, thereby promoting industrial-scale process efficiency.

**Figure 5:**
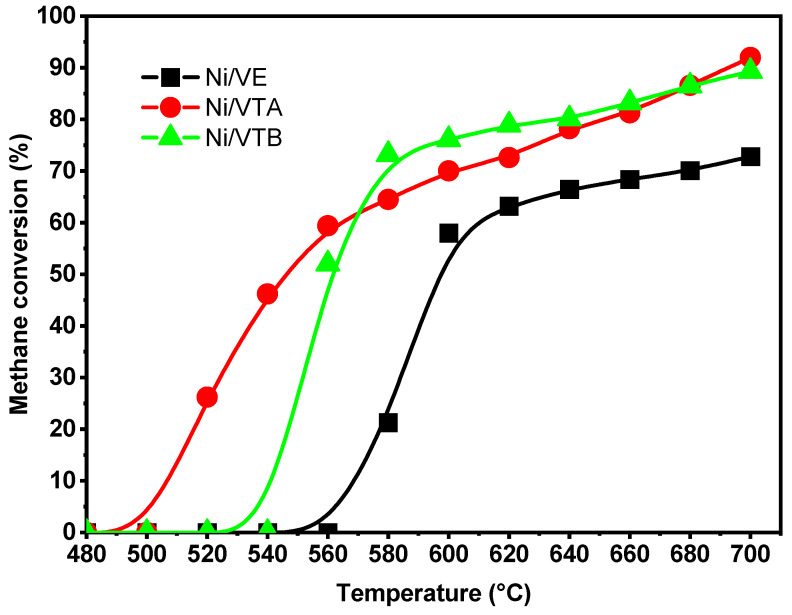
Methane conversions (%) of Ni/VE, Ni/VTA, and Ni/VTB catalysts depending on the temperature of reaction.

**Figure 6:**
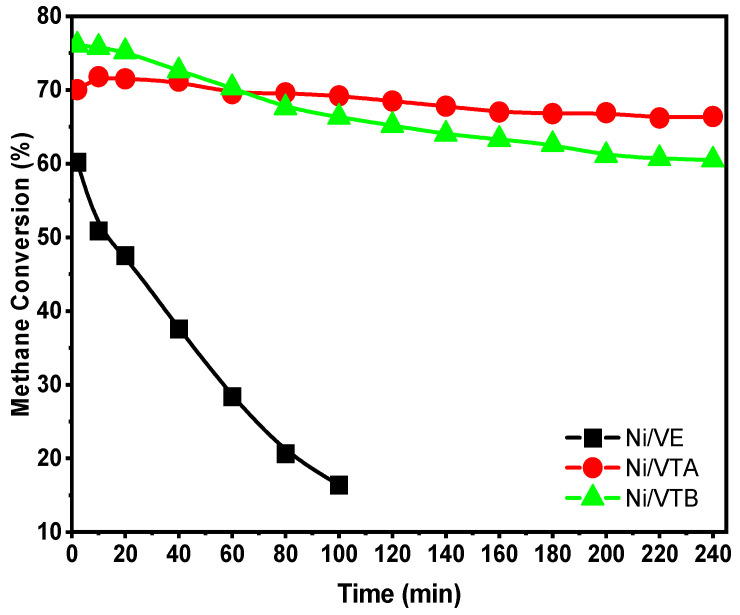
Methane conversions (%) on Ni/VE, Ni/VTA, and Ni/VTB catalysts depending on the reaction time.

**Figure 7:**
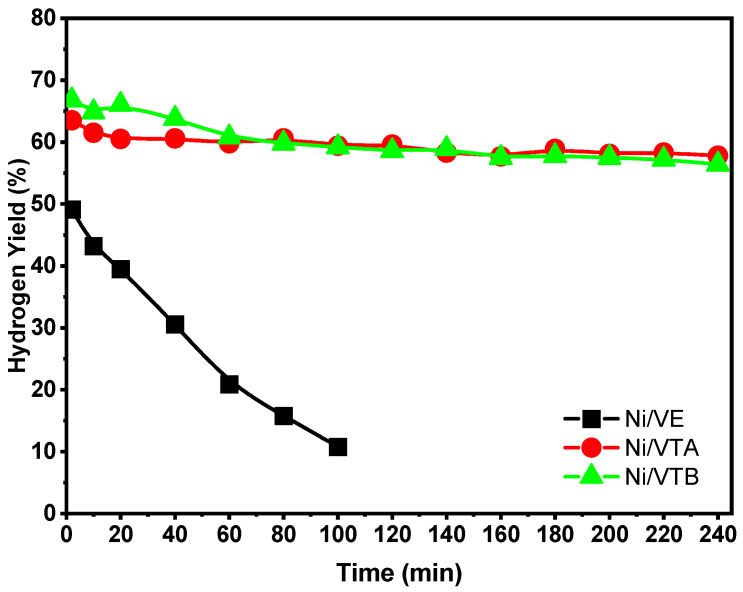
Hydrogen yields (%) over Ni/VE, Ni/VTA, and Ni/VTB catalysts as function of reaction time.

Ni-based catalysts supported on expanded vermiculite and vermiculite exposed to HCl or NaOH solutions were tested for dry methane reforming with CO_2_ at low temperatures (Figure 5, Figure 6 and Figure 7). Methane activation initiates at different temperatures for each catalyst: 520 °C for Ni/VTA, 560 °C for Ni/VTB, and 580 °C for Ni/VE, as shown in Figure 5. As the temperature increases, a corresponding rise in CH_4_ conversion was observed, reaching 93% for the Ni/VTA catalyst at 700 °C. In comparison, Ni/VTB and Ni/VE only achieved 89% and 72% conversion, respectively, at the same temperature. This phenomenon is closely related to the endothermicity of the reaction [27]. The activity of the Ni/VTA and Ni/VTB catalysts appeared to be significantly influenced by the type of treatment at temperatures lower than 620 °C, while it seemed to be unaffected in the range of 640–700 °C. Nevertheless, a substantial effect on catalytic performance between untreated expanded vermiculite and acid- or base-treated vermiculite was observed.

Catalytic stability under operating conditions is a critical factor. The CH_4_ conversions and H_2_ yields plotted against time at 600 °C over Ni/VTA, Ni/VTB, and Ni/VE for 240 min are shown in Figure 6 and Figure 7. The Ni-supported raw vermiculite exhibits poor catalytic activity and undergoes rapid deactivation during dry methane reforming. After only 100 min on stream, the CH_4_ conversion and H_2_ yield over Ni/VE decreased significantly from 60% to 16% and from 49% to 10%, respectively. Figure 6 and Figure 7 show that the methane conversion reduced from 76% to 61% for the Ni/VTB catalyst, while the hydrogen yield decreased slightly from 65% to 57%. Similarly, the yields from the conversions of methane and hydrogen slightly varied from 72% to 67% and from 62% to 58%, respectively, after a functioning time of 240 h for Ni/VTA. It is evident that the catalytic activity gradually decreased for all of the catalysts as the duration of the tests increased. The CH_4_ conversion only decreased by 5% and 15% for Ni/VTA and Ni/VTB, respectively, during 240 min on stream. This indicates that the acid treatment considerably enhanced the catalytic activity of employed catalysts, which correlates with their easy reducibility at low temperatures.

#### 2.2.2. Spent Catalysts Characterization (TGA-TDA and XRD)

**Figure 8:**
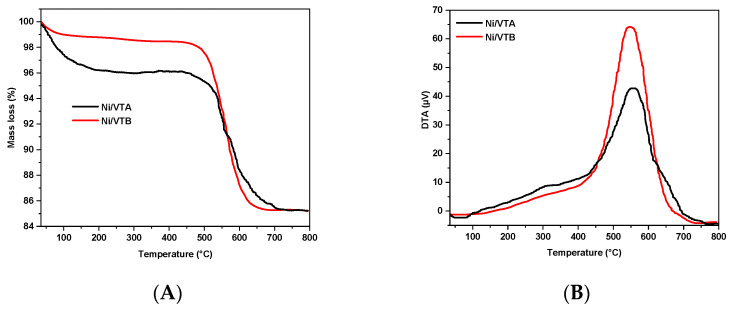
TGA profiles (**A**) and DTA profiles (**B**) of Ni/VTA and Ni/VTB spent catalysts.

Thermogravimetric Analysis (TGA-TDA) determined the type and quantity of carbon deposition on the Ni/VTA and Ni/VTB catalysts after 240 min of reaction at 600 °C in air, from ambient temperature to 800 °C (Figure 8A,B). The carbon deposition is mainly caused by both CH_4_ decomposition and CO disproportionation reactions, which represent favorable conditions for CH_4_/CO_2_ reforming. The TGA profile of the Ni/VE catalyst indicates no mass loss, which agrees with the low activity of the catalyst.

The TGA results (Figure 8A) show that the Ni/VTA and Ni/VTB spent catalysts exhibited some weight loss over the entire investigated 30 to 800 °C range. The initial weight losses observed at around 100–150 °C for Ni/VTA and Ni/VTB are approximately 3.4% and 1.3% *w*/*w*, respectively, which can be attributed to the elimination of water [28,29,30]. No mass changes occurred between ~150–450 °C up to a sudden weight change at 500 °C. Coke gasification is the reason for the noticeable weight loss between 500 and 700 °C, as coke was oxidized to CO and CO_2_.

It is interesting to note that the percentage of carbon deposited onto the Ni/VTA catalyst (10.5%) is lower than the amount deposited onto the Ni/VTB catalyst (13.1%).

DTA profiles in Figure 8B indicate the presence of a single intense exothermic peak for both catalysts located between 450 and 700 °C. The Ni/VTA and Ni/VTB catalysts exhibited maximum peak temperatures of 557 °C and 548 °C, respectively. The presence of this particular type of carbon results from the oxidation of carbon filaments (carbon nanotubes), according to S. Damyanova et al. [31] and W.D. Zhang [32].

**Figure 9:**
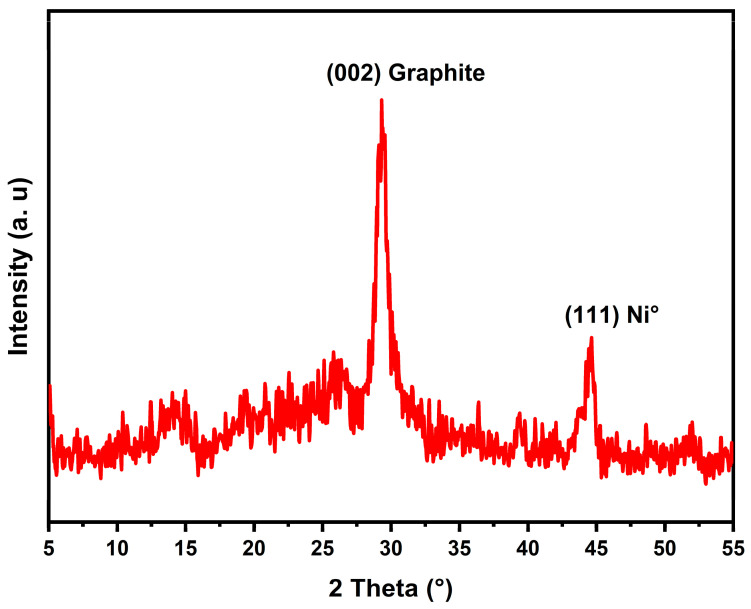
XRD pattern of Ni/VTA spent catalyst after DRM.

To examine the species of carbon formed, XRD analysis was carried out on the spent Ni catalyst deposited on the acid-treated support after DRM (Figure 9). The appearance of two closely spaced lines at approximately 2θ = 30° and 45° in the diffractogram indicates the formation of graphitic carbon on the catalyst surface for the first line (002), while the second line (111) corresponds to the reduced Ni [33,34].

**Figure 10:**
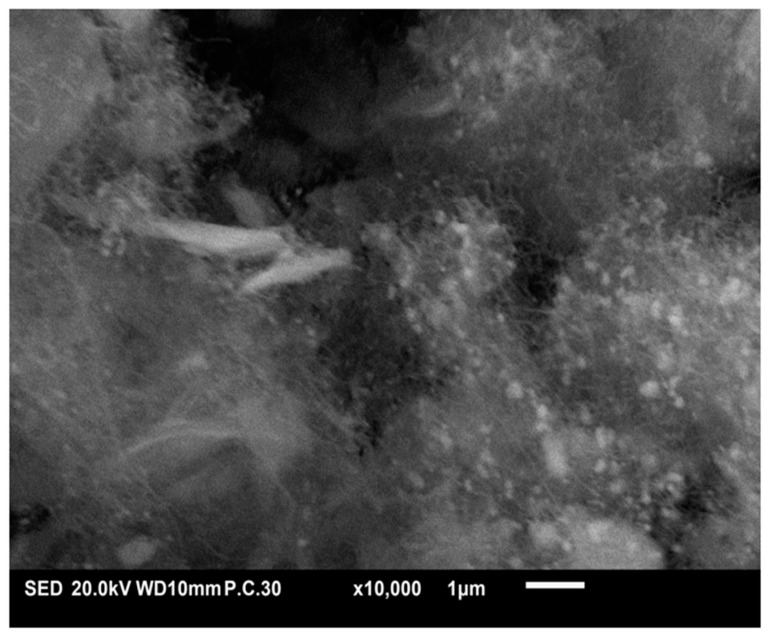
SEM image of Ni/VTA spent catalyst after DRM.

In addition, the formation of graphitic filamentous carbon was confirmed by SEM analysis (Figure 10) of a portion of the spent catalyst.

#### 2.2.3. Ethanol Steam Reforming

**Figure 11:**
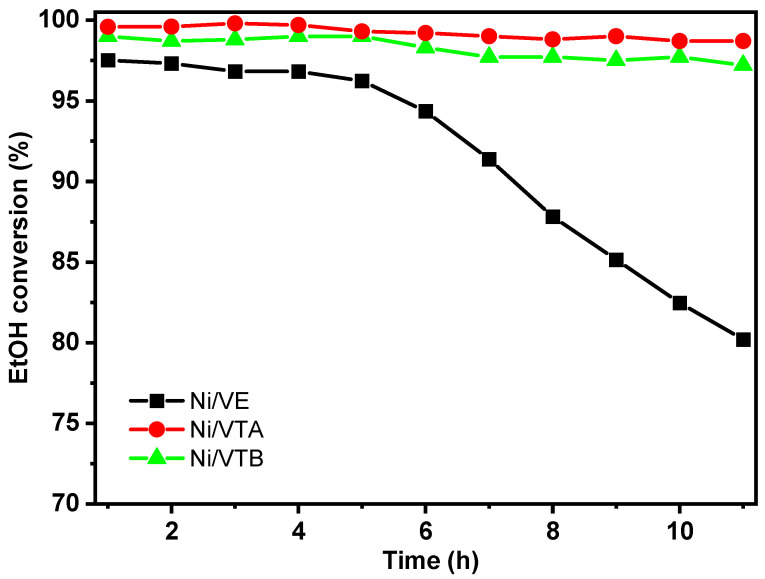
Ethanol conversion rates vs. time over Ni/VE, Ni/VTA, and Ni/VTB catalysts.

For a more detailed analysis and to further assess the stability of the prepared catalysts, some experiments were conducted in ESR for a period of 10 h at 600 °C, using a W/F of 18 g_cat_ (Figure 11). It was observed that the ethanol conversions for all catalysts are significantly high, starting from the first hour of reaction, ranging from approximately 98% to 100%. However, there are notable differences in the behavior of the catalysts based on the type of vermiculite support used. The samples prepared by acid or base treatments exhibited stable activity, presenting only a slight deactivation compared to that prepared using the support from the basic treatment [35]. In contrast, the catalyst produced from untreated vermiculite displayed a different pattern. It started with similar activity of the other catalysts, then rapidly deactivated after 5 h of reaction. As is commonly reported in reforming reactions, catalyst deactivation typically stems from two primary factors: carbon formation and sintering on the catalyst [36,37,38,39].

**Figure 12:**
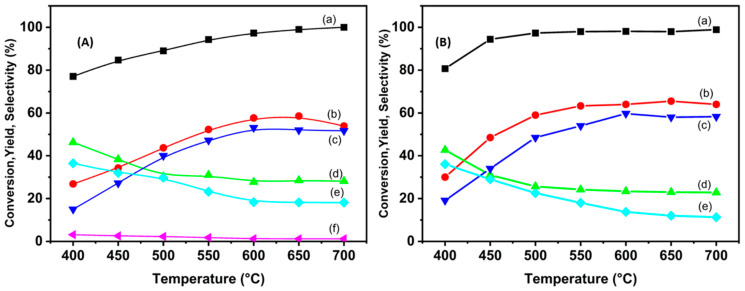
(a) Ethanol conversion rates versus temperature, (b) hydrogen yields, and (c) CO_2_, (d) CO, (e) CH_4_, and (f) C_2_H_6_ selectivities over the catalysts (**A**) Ni/VTB and (**B**) Ni/VTA.

In summary, the results underscore the impressive initial performance of the catalysts in achieving high ethanol conversion rates. Particularly noteworthy is the early success of the Ni/VE catalyst, followed by its subsequent deactivation. The observed catalyst deactivation after 5 h highlights the importance of further investigation and mitigation of issues related to carbon formation and sintering for ensuring long-term catalyst stability and efficiency. However, it is important not to overlook the sintering effects caused by the presence of various cations, which are eliminated through acid or base washing [40]. Considering the instability observed in the Ni/VE sample, this study proceeds with the samples obtained from acid or base pre-treated vermiculite. Figure 12A,B illustrate the impact of temperature on various reaction products evolution.

The catalytic activity of both catalysts shows a significant correlation with increasing reaction temperature. For the Ni/VTA catalyst, ethanol conversion approaches near completion at 500 °C (Figure 12B), whereas the highest ethanol conversion rate for the Ni/VTB catalyst is achieved at 700 °C. At low temperature (400 °C), product formation such as ethane, ethylene, and acetaldehyde (C2) are observed. This is attributed to the relatively weak C–C bond-breaking ability in the presence of nickel [41,42,43]. The emergence of acetaldehyde can be attributed to the dehydrogenation of ethanol [44]. Moreover, acetaldehyde is recognized as an intermediate product in ethanol reforming, capable of either decomposing into CO and CH_4_ through C–C bond cleavage, or transforming into ethane and water through C–O cleavage [45].

**Figure 13:**
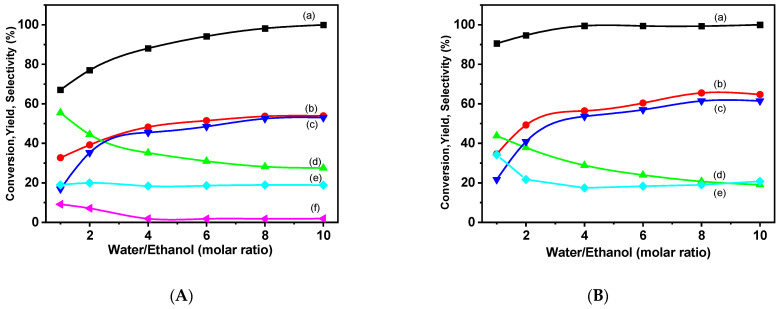
(a) Ethanol conversion rates versus Water/Ethanol molar ratio, (b) hydrogen yields, and (c) CO_2_, (d) CO, (e) CH_4_, and (f) C_2_H_6_ selectivities over the catalysts (**A**) Ni/VTB and (**B**) Ni/VTA.

Figure 13 presents the ethanol conversion data over Ni/VTB and Ni/VTA as a function of the water/ethanol ratio. In the case of the two catalysts under examination, Ni/VTB (Figure 12A) and Ni/VTA (Figure 12B), the ethane conversion rate displays an increase as the S/E molar ratio rises, reaching a plateau at a ratio of 9.

Additionally, there is a simultaneous increase in H_2_ yield and selectivity for CO_2_ in both catalysts, accompanied by a gradual decrease in selectivity for CO and CH_4_ for Ni/VTA, while methane selectivity remains almost stable for Ni/VTB. The presence of an adequate amount of water is associated with an elevation in both the ethanol conversion rate and H_2_ yield. This outcome is attributed to the well-established notion that the presence of water generally promotes reforming reactions [46].

Furthermore, upon comparing the performance of the two catalysts, it becomes evident that the catalyst supported by vermiculite and treated with an acid solution outperforms the other. It is essential to highlight that this treatment results in the removal of a portion of the ions situated within the interlayer space. This variance in performance between the two catalysts may be attributed to the different reducibilities between them. Notably, in terms of hydrogen production, the acidic catalyst demonstrates relatively superior performance.

**Figure 14:**
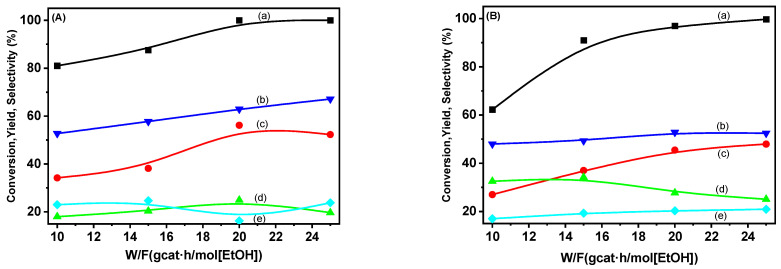
(a) Ethanol conversion rates depending on space velocity (W/F), (b) hydrogen yields, and (c) CO_2_, (d) CO, and (e) CH_4_ selectivities over the catalysts (**A**) Ni/VTB and (**B**) Ni/VTA.

The influence of space velocity (W/F) on the ethanol conversion rate and product distribution was investigated across a range of space velocities, expressed as ratio g_cat_ h/mol [EtOH] and varying from 10 to 25, at a constant temperature of 600 °C, while maintaining a molar W/E ratio of 10. As illustrated in Figure 14, the ethanol conversion rate for both studied catalysts exhibited an upward trend, especially for ethanol conversion, by decreasing the space velocity, although even at high space velocity values, i.e., at low g_cat_ h/mol [EtOH] ratio, the conversion values, neither the hydrogen yields, didn’t decrease significantly. 

## 3. Experimental

### 3.1. Catalyst Preparation

All chemicals employed in this study were of analytical quality (sourced from Aldrich (Burlington, MA, USA) and Fluka (Tokyo, Japan)) and were employed without additional refinement. The raw expanded vermiculite (VE) employed in this research was provided by Xinjiang Yuli Xinlong Vermiculite Co., Ltd., located in Xinjiang, China. The chemical composition of the studied vermiculite is mainly 39.78% SiO_2_, 22.84% MgO, 20.14% Al_2_O_3_, 6.12% K_2_O, 4.82% Fe_2_O_3_, 3.14% CaO, 2.12% Na_2_O, and 0.87% TiO_2_.

The acid-treated sample (VTA) was prepared by leaching vermiculite for 2 h at 80 °C with HCl solution (1 M), using a ratio of 1 g/10 cm^3^ between the clay mineral mass and the acid solution. The base-treated support (VTB) was obtained using NaOH solution (1 M) for 24 h at 60 °C. The obtained VTA and VTB samples were filtered, washed, and dried at 120 °C, then calcined at 700 °C for 12 h in air.

The preparations of loaded nickel catalysts from acidic and basic treatments and untreated, named Ni/VTA, Ni/VTB, and Ni/VE, were carried out using the wetness impregnation method, with a Ni loading of 15 wt.%. The impregnated materials were dried for 12 h at 120 °C, then calcined at 700 °C for 24 h in air.

### 3.2. Characterization Methods

Microwave plasma atomic emission spectroscopy (ICP-AES) with an Agilent 4200 spectrometer (Santa Clara, CA, USA) was used to determine the elemental composition of the catalysts.

The specific surface area, pore volume, and pore size distribution of the samples were measured at −196 °C with a nitrogen sorption technique using ASAP 2020 equipment (Micromeritics, Norcross, Catalysts 2023, 13, 606 15 of 18 GA, USA). The powder samples (approximately 200 mg) were degassed for 2 h at 250 °C prior to measurement. The specific surface area was estimated with the Brunauer–Emmett–Teller (BET) method at a standard pressure ranging from 0.05 to 0.3 P/P_0_. The pore volume and pore size distributions were determined by analyzing the desorption zone according to the Barrett, Joyner, and Halenda (BJH) computational method.

XRD patterns were carried out on a Siemens D 5000 high-resolution diffractometer with a copper anticathode (Kα = 1.5406 Å). With a scan step of 0.01°, the data were collected over a 2θ range from 5° to 90°. The Scherrer Equation was used to confidently calculate the particle size:

(1) $$D_{hkl}=\frac{K\lambda }{bcos\theta }$$

FTIR spectra were recorded on a Perkin-Elmer 1600 spectrometer (Waltham, MA, USA) with self-supporting KBr discs at room temperature in the range 400 to 4000 cm^−1^.

Profiles of temperature programmed reduction (TPR) were registered under atmospheric conditions in a microreactor packed with approximately 100 mg of oxidized catalyst. The samples were reduced at a total flow rate of 62.5 cm^3^ min^−1^ with a heating rate of 10 °C min^−1^, using a 2.5:60 volume ratio of H_2_/He mixture. A mass spectrometer was used to monitor hydrogen consumption.

The microstructure analysis of catalysts was performed using a scanning electron microscope (SEM) (Thermo Ficher (Norristown, PA, USA)—Quanta 650 environmental) with an energy-dispersive X-ray spectroscopy (EDS) detector in backscattered electron (BSE) mode. A beam voltage of 20 kV was used during imaging. A Mettler Toledo TGA/DSC Star system (Columbus, OH, USA) was used for thermogravimetric/differential thermal analysis (TGA/DTA). A mass quadrupole (Thermostar^TM^, Balzers, Liechtenstein) was used for on-line monitoring of the evolution of gaseous species.

### 3.3. Catalytic Tests

#### 3.3.1. Methane Reforming Reaction

In a continuous flow quartz microreactor maintained at atmospheric pressure, the methane reforming reaction was examined. Before starting the reaction, 100 mg of the catalysts was sieved to a particle size of 120–180 µm and placed in the reactor between two quartz wool plugs.

The reaction temperature was ramped up from ambient to 700 °C, applying a heat rate of 5 °C per minute. A mixture of feed composed of CH_4_/CO_2_/N_2_ in a ratio of 2:2:56 was placed in the reactor under a total flow reaching 60 mL min^−1^. It is important to note that all catalysts were employed without prior reduction with hydrogen.

Particular emphasis was placed on assessing the stability of the catalysts over the course of the reaction. Hydrocarbons produced during the reaction were analyzed in real-time using an on-line chromatograph (Delsi) with the column type Porapak Q and a flame ionization detector (FID). A second gas chromatograph (Shimadzu, Kyoto, Japan, 8A) connected to a thermal conductivity (TC) detector with a carbosphere column and molecular sieve columns (5A) was used to determine carbon dioxide and hydrogen concentrations.

The conversion of methane and carbon dioxide, as well as the hydrogen yield of the samples, was obtained from the following equations:

(2) $${CH}_{4}\;conversion\left(\%\right)=\frac{{\left({CH}_{4}\right)}_{in}-{\left({CH}_{4}\right)}_{out}}{{\left({CH}_{4}\right)}_{in}}\times 100$$

(3) $${CO}_{2}\;conversion\left(\%\right)=\frac{{\left({CO}_{2}\right)}_{in}-{\left({CO}_{2}\right)}_{out}}{{\left({CO}_{2}\right)}_{out}}\times 100$$

(4) $$H_{2}\;yield\left(\%\right)=\frac{{\left(H_{2}\right)}_{out}}{{\left({CH}_{4}\right)}_{in}}\times \frac{100}{2}$$

(5) $$CO\;yield\left(\%\right)=\frac{{CO}_{out}}{{\left({CH}_{4}\right)}_{in}+{\left({CO}_{2}\right)}_{in}}\times 100$$

#### 3.3.2. Ethanol Steam Reforming (ESR)

The catalyst’s performance in the ethanol steam reforming (ESR) process was assessed using a quartz tube fixed-bed reactor. Prior to each test, the catalyst underwent in situ treatment at 700 °C in a 1:8 (*v*/*v*) H_2_:N_2_ stream at a flow rate of 60 mL min^−1^ for one hour. Following this pre-treatment, the desired water/ethanol ratio was introduced into the evaporator at 150 °C using a syringe pump, while an N_2_ flow at a rate of 20 mL min^−1^ facilitated vapor transport into the reactor.

The investigation confidently focused on the conversion of ethanol and the production of gas. In each test, a fresh catalyst was used for a duration of 6 h. The gas products recovered from the outlet of the reactor were analysed on-line using a Perichrom PR2100 gas chromatography (GC) instrument (Paris, France) controlled by Winilab software (III 5.0).

For reactants and products analysis, two detectors were employed: a thermal conductivity detector (TCD) equipped with a TDX-01 column to measure concentrations of H_2_, CO_2_, and CO; a flame ionization detector (FID) equipped with a Porapak-Q column was utilized to identify potential organic species, including CH_4_, C_2_H_4_, C_2_H_6_, CH_3_CHO, and CH_3_COCH_3_. Unreacted ethanol was examined offline via FID. The key performance parameters, including ethanol conversion (X_EtOH_), hydrogen yield (Y_H2_), and the distribution of gaseous carbon-containing products (CH_4_, CO, CO_2_, C_2_H_4_, C_2_H_6_, CH_3_CHO, and CH_3_COCH_3_), were determined as follows:

(6) $$X_{EtOH}\left(\%\right)=\frac{{EtOH}_{in}-{EtOH}_{out}}{\left({EtOH}_{out}\right)\times 100}$$

(7) $$Y_{EtOH}\left(\%\right)=\frac{moles\;H_{2}\;produced}{\left(6x\;moles\;EtOH\;converted\right)}\times 100$$

(8) $$Si\left(\%\right)=\frac{mole\;of\;gaseous\;product\;i}{\left(mole\;all\;gaseous\;products\right)}\times 100$$

## 4. Conclusions

This study investigated the use of vermiculite as a support material for nickel catalysts in two key processes for syngas production: dry reforming of methane with CO_2_ and steam reforming of ethanol. The vermiculite underwent either acid or base treatment, followed by the preparation of Ni catalysts via incipient wetness impregnation. After acid (Ni/VTA) or base (Ni/VTB) treatment, vermiculite became an effective support for nickel in dry methane reforming. Acidic treatment notably enhanced the reduction of nickel species and lowered the amount of carbon formed after dry reforming, with respect to the Ni sample deposited over the alkali-treated oxide. The prepared catalysts were also evaluated in ethanol steam reforming under various conditions including temperature, water/ethanol ratio, and space velocity in terms of different ratio g_cat_ h/mol [EtOH], both catalysts exhibited good performance maintaining relatively high conversion values and hydrogen yield at the high space velocity. 

## Figures and Tables

**Table 1:** Specific surface area, pore volume, and chemical composition of Ni over the raw and treated vermiculite.

Catalyst	SSA	Pore Volume	Chemical Composition Mass (%) by ICP Analysis								
	(m^2^ g^−1^)	(cm^3^ g^−1^)	Ni	SiO_2_	MgO	Al_2_O_3_	Fe_2_O_3_	K_2_O	CaO	Na_2_O	TiO_2_
Ni/VE	36	0.10	15.0	33.8	19.8	17.1	2.8	5.1	3.2	2.1	1.1
Ni/VTB	38	0.11	15.0	36.4	18.6	16.6	2.6	4.9	2.9	2.0	1.0
Ni/VTA	270	0.55	15.0	78.4	2.8	2.6	0.3	0.5	0.2	0.1	0.1

## Data Availability

Data are contained within the article.
